# A Case of Hydroxocobalamin-Induced False Blood Leak Alarm on Dialysis Machine

**DOI:** 10.1177/2324709619883466

**Published:** 2019-11-08

**Authors:** Praveen Datar, Jasdeep Singh Sidhu, Jeevanjot Virk, Osama Mukhtar, Frances Schmidt, Vijay Gayam

**Affiliations:** 1 Interfaith Medical Center, Brooklyn, NY, USA

**Keywords:** cyanide poisoning, hydroxocobalamin, hemodialysis

## Abstract

Hemodialysis machines are equipped with a blood leak detector/alarm to prevent loss of blood following rupture of semipermeable membrane; the blood leak alarms could also be triggered by sensor malfunction or presence of air bubbles in the system. Hydroxocobalamin is a Food and Drug Administration–approved rapid-acting antidote to cyanide poisoning that converts cyanide to nontoxic cyanocobalamin. Side effects are reddish discoloration of skin and body fluids, urticarial rash, and rarely anaphylaxis. In this article, a case of false blood leak alarm following treatment of cyanide poisoning with hydroxocobalamin is reported, wherein the blood leak detector in dialysis machines prevented the patient from undergoing hemodialysis by repeatedly activating blood leak alarms. Continuous renal replacement therapy was used to overcome this problem. As the use of hydroxocobalamin increases, health care professionals should be educated about its potential to interfere with hemodialysis.

## Introduction

Carbon monoxide and cyanide poisoning usually occur simultaneously in victims of smoke inhalation. Cyanide poisoning is seen most commonly following exposure to fires. The usual culprit for cyanide poisoning is inhaled hydrogen cyanide, which is produced by incomplete combustion of many household items. The treatment for cyanide poisoning includes basic life support with 100% oxygen, assisted ventilation (if the patient is unconscious or has airway compromise), decontamination, vitals and metabolic support, and the use of an antidote. One of the known and fast-acting antidotes for cyanide poisoning is hydroxocobalamin, which converts cyanide ions into cyanocobalamin (vitamin B_12_), which is nontoxic.^1^ It has been approved by the Food and Drug Administration for use in cyanide toxicity.^2^ Its known side effects include reddish discoloration of skin and body fluids, urticarial rash, and rarely anaphylaxis.^3,4^

Hemodialysis machines are designed to maintain a barrier between blood and dialysate to maintain sterility and prevent blood loss.^5^ On detecting presence of blood in dialysate fluid, the blood leak sensors activate and set an alarm and the process of dialysis also stops.^6^ Blood leak can occur due to mechanical damage to the semipermeable membrane between dialysate and blood flow, or there could be false alarm in certain situations where the blood leak sensors are triggered despite no real leakage such as presence of air bubbles or damage to sensors.^7^

In this article, a case of false blood leak alarms secondary to hydroxocobalamin treatment for cyanide poisoning is described, in which repeated activation of blood leak detector prevented the patient from undergoing an acute hemodialysis treatment following administration of hydroxocobalamin for cyanide poisoning.

## Case Report

A 73-year-old male with past medical history of end-stage renal disease and chronic obstructive pulmonary disease was brought to our emergency department after a house fire. The fire apparently started after an electrical short circuit in his room, awakening him and his wife from their sleep. He was exposed to significant smoke inhalation and later lost consciousness. Emergency medical services was notified, and the patient was rescued from the house after 20 minutes of exposure to smoke and heat.

Vitals in the field were as follows: blood pressure 90/45 mm Hg, oxygen saturation 100% on room air with a carbon monoxide level of 49. Following the rescue, initial high-flow oxygen therapy was started by the ambulance service. With strong suspicion of concomitant cyanide poisoning, sodium thiosulfate and hydroxocobalamin was administered en route to the emergency department.

On arrival, triage vitals showed blood pressure 80/50 mm Hg, heart rate 72 beats per minute, and saturation of 95% on ambu-bag. Initial general physical examination revealed cherry red skin all over the body of the patient, with strawberry-colored urine draining through the Foley catheter. There was no evidence of external burns, singed nostril hair, stridor, nor evidence of airway obstruction. The Glasgow Coma Scale on arrival was 3/15. He was immediately intubated and started on mechanical ventilation (pressure-regulated volume control mode with 100% oxygen). Subsequently, arterial blood gas analysis revealed pH 6.766 (reference range [RR] = 7.350-7.450), pO_2_ 352 mm Hg (RR = 75-100 mm Hg), pCO_2_ 58.3 (RR = 35-45 mm Hg), and base excess of −23.9 (RR = −3 to 3). Carboxyhemoglobin level was 25% (RR = 0.0% to 1.5%). Patient was given fluid resuscitation with 0.9% normal saline, and inotropic support was started with norepinephrine. Hydroxocobalamin was readministered. Acidosis improved with improvement in pH from 6.77 to 7.2 (RR = 7.5-7.45), and lactic acid trended down from 22 to 1.1 (RR = 0.5-1.9). Carboxyhemoglobin also trended down from 49 to 2.7 (RR = 0.0-1.5), but it could have been falsely decreased due to cyanocobalamin administration. Patient’s blood pressure normalized, and vasopressors were discontinued. Bronchoscopy was done, which revealed erythematous mucosa and black streaks and tar-like fragments on the bronchial walls. Pink-colored lavage mixed with soot was suctioned.

Hemodialysis was planned but it could not be continued as the hemodialysis machine started triggering the “blood leak” alarm, which persisted despite changing the hemodialysis machines multiple times. It was suspected that due to the chromogenic properties of hydroxocobalamin in the patient’s system, the red discoloration of body fluids, which was also witnessed in other body fluids including the urine and tracheal aspirate, activated the blood leak alarm and prevented intermittent hemodialysis. Under these circumstances, renal replacement therapy was planned for the patient. A NxStage System One machine was used for the continuous veno-venous hemodialysis (CVVHD). The patient was later successfully liberated from the respirator.

## Discussion

Hydroxocobalamin has been classically used in treatment of cyanide poisoning to convert cyanide into much less toxic cyanocobalamin by replacing the hydroxo ligand of the compound hydroxocobalamin.^1^ One of the known side effects of hydroxocobalamin is dark red discoloration of body fluids, as seen in our case, which may interfere with urine and serum colorimetric analysis.^3,4^ Red-purple discoloration of the urine can be noted up to 35 days of administration of hydroxocobalamin.^4^ However, in general, hydroxocobalamin is considered a safe antidote for cyanide poisoning.^2,8^

The inability to perform intermittent hemodialysis was one of the most significant challenges for us in management of this patient as our patient had end-stage renal disease and was on scheduled intermittent hemodialysis. Normally, in the hemodialysis machine, the blood and the dialysate pass in counter-current fashion across a semipermeable membrane for effective diffusion of solutes. All the hemodialysis machines have a built-in infrared or photodetector sensor activated by presence of red blood cells in the dialysate fluid and that cause the dialysis machine to stop working on its activation to prevent any blood loss during dialysis.^5^ This alarm system is made up of a photodetector and a light source set up across the dialysate column with the light source illuminating toward the photodetector that is meant to detect changes in transparency of the dialysate fluid expected in blood leak. Actual blood leak can occur due to mechanical damage to the semipermeable membrane between dialysate and blood flow. The blood leak alarm can also be triggered despite no real leakage due to presence of air bubbles, damage to sensors, hemolysis, or with old/reused dialysate membrane.^9^ Chromogenic substances such as hydroxocobalamin can also lead to discoloration of dialysate fluid, activating the blood leak detector and causing the dialysis machine to shut down.^7^

The Fresenius 2008K dialysis machine employs a 2-color transmitter with red and green light, and following a leak, the green light is absorbed by the blood leading to the activation of the detector.^6,10^ On the other hand, NxStage machine and Prisma Flex machines have a single optical emitter that emits infrared wavelength, and the detector reads the dispersion of this light. As this light source emits light beyond visible spectrum and does not rely on absorption of a particular wavelength, it is not affected by the discoloration of the dialysate fluid.^11^ Gambro Phoenix X36, NxStage, and Prisma Flex dialysis machines are reported to be not affected by this dialysate discoloration due to hydroxocobalamin and can be safely used for dialysis in such patients.^8,11,12^

In our case, internal alarms were repeatedly activated due to detection of pseudo blood leak malfunction of the Fresenius 2008K hemodialysis following which 2 sessions of scheduled hemodialysis were cancelled causing volume overload state and delayed his weaning from the ventilator. We started the patient on CVVHD using a NxStage System One machine, and he was successfully liberated from the respirator. There is no definite time limit for transition back to intermittent hemodialysis as serum hydroxocobalamin level is noticeable up to 30 days following its administration.^7^ In our case, the patient was successfully transitioned back to intermittent hemodialysis after 7 days of CVVHD.

As hydroxocobalamin is being used more and more frequently for management of suspected cyanide poisoning every year, health care providers may come across these challenges more frequently.^13,14^ In our understanding, it is important to have increased awareness among emergency room physicians, critical care physicians, and nephrologists about hydroxocobalamin-induced false blood leak alarms during intermittent hemodialysis with certain dialysis machines and the need to timely utilize continuous renal replacement therapy with dialysis machines from a different manufacturer to limit the morbidity and mortality associated with failed hemodialysis therapy.
